# Contributions of the default mode and central executive networks during posterior cingulate cortex-targeted fMRI neurofeedback in PTSD

**DOI:** 10.1016/j.nicl.2025.103891

**Published:** 2025-10-22

**Authors:** Jonathan M. Lieberman, Ruth A. Lanius, Maria Densmore, Jean Théberge, Daniela Rabellino, Paul A. Frewen, Frank Scharnowski, Rakesh Jetly, Benicio N. Frey, Fardous Hosseiny, Tomas Ros, Andrew A. Nicholson

**Affiliations:** a Department of Psychiatry and Behavioural Neurosciences, McMaster University, Hamilton, Ontario, Canada; b Imaging, Lawson Research Institute, London, Ontario, Canada; c Atlas Institute for Veterans and Families, Ottawa, Ontario, Canada; d Department of Neuroscience, Western University, London, Ontario, Canada; e Department of Psychiatry, Western University, London, Ontario, Canada; f Department of Psychology, Western University, London, Ontario, Canada; g Department of Cognition, Emotion, and Methods in Psychology, University of Vienna, Vienna, Austria; h Department of Medical Biophysics, Western University, London, Ontario, Canada; i Department of Diagnostic Imaging, St. Joseph’s Health Care London, London, Ontario, Canada; j The University of Ottawa Institute of Mental Health Research, Royal Ottawa Hospital, Ottawa, Ontario, Canada; k Mood Disorders Treatment and Research Clinic, St. Joseph’s Healthcare Hamilton, Ontario, Canada; l Department of Neuroscience and Psychiatry, University of Geneva, Geneva, Switzerland; m School of Psychology, University of Ottawa, Ottawa, Ontario, Canada

**Keywords:** Post-traumatic Stress Disorder, fMRI Neurofeedback, Posterior Cingulate Cortex, Independent Component Analysis, Default Mode Network, Central Executive Network

## Abstract

**Introduction:**

Functional magnetic resonance imaging-based neurofeedback (fMRI-NFB) enables individuals to regulate brain activity implicated in psychopathology, including post-traumatic stress disorder (PTSD). While most fMRI-NFB studies in PTSD target the amygdala, which engages the central executive network (CEN) for top-down regulation, the posterior cingulate cortex (PCC), a core node of the default mode network (DMN), has recently emerged as a promising therapeutic target. However, the relative contributions of intra-network DMN mechanisms versus inter-network CEN engagement during PCC downregulation remain unclear.

**Methods:**

We used independent component analysis (ICA) to examine DMN and CEN functional connectivity during PCC-targeted fMRI-NFB in individuals with PTSD (n = 14) and healthy controls (n = 15) while viewing trauma-related/distressing words. Mixed-design repeated measures ANOVAs assessed within- and between-group connectivity changes, and clinical correlations were explored.

**Results:**

During PCC downregulation, PTSD participants exhibited greater DMN connectivity than controls with trauma-related regions, including the precentral gyrus and anterior insula, which correlated with PTSD severity and emotion regulation difficulties. Conversely, CEN connectivity decreased in both groups, with PTSD participants showing progressively reduced connectivity across training. Direct comparisons revealed that DMN connectivity exceeded CEN connectivity with several brain regions, particularly among PTSD participants.

**Conclusion:**

These findings highlight the predominant role of DMN mechanisms in PCC downregulation in PTSD. The reliance on intra-network DMN processes over CEN-driven regulation underscores distinct network dynamics that may be unique to PCC-targeted fMRI-NFB. These neural mechanistic insights may inform targeted fMRI-NFB protocols to recalibrate altered DMN connectivity and enhance emotion regulation in PTSD.

## Highlights

• PTSD participants had greater DMN connectivity than controls with trauma regions.
• Increased DMN connectivity was positively correlated with clinical measures.
• CEN connectivity decreased, with PTSD participants showing progressive reductions.
• PCC-targeted fMRI-NFB primarily engages DMN rather than CEN-driven regulation.

## 1 Introduction

Post-traumatic stress disorder (PTSD) is a complex and prevalent mental health condition often marked by chronic and debilitating symptoms following trauma exposure. These symptoms span multiple domains, including intrusive experiences (e.g., memories and physiological reactions), avoidance of trauma-related stimuli, negative changes in cognition and mood, heightened arousal and reactivity, and dissociative symptoms such as depersonalization and derealization (American Psychiatric Association, 2022). Existing psychotherapies and pharmacotherapies for PTSD face significant challenges, with one-half to two-thirds of patients failing to achieve complete remission (Bradley et al., 2005, Haagen et al., 2015, Krystal et al., 2017, Ravindran and Stein, 2009, Stein et al., 2006). Furthermore, dropout rates remain high, particularly during trauma-focused interventions (Bisson et al., 2013, Goetter et al., 2015, Kehle-Forbes et al., 2016, Lewis et al., 2020). Together, these limitations have driven growing interest in exploring novel, neurobiologically informed treatment approaches such as real-time fMRI neurofeedback (fMRI-NFB).

fMRI-NFB is an emerging technique that enables individuals to non-invasively self-regulate brain activity associated with psychopathology (Sitaram et al., 2017). It has been deployed in the context of various psychiatric disorders (e.g., Hamilton et al., 2016, Li et al., 2013, Linden et al., 2012, Mehler et al., 2018, Young et al., 2017), including PTSD (Chiba et al., 2019, Gerin et al., 2016, Lieberman et al., 2023a, Lieberman et al., 2023b, Lieberman et al., 2025, Misaki et al., 2018b, Misaki et al., 2019, Misaki et al., 2021, Nicholson et al., 2016, Nicholson et al., 2018, Nicholson et al., 2021, Weaver et al., 2020, Zhao et al., 2023, Zotev et al., 2018, Zweerings et al., 2018, Zweerings et al., 2020). Recent research suggests that fMRI-NFB may be an effective approach for restoring neural disruptions and reducing associated symptoms in PTSD (Chiba et al., 2019, Gerin et al., 2016, Lieberman et al., 2023a, Lieberman et al., 2023b, Misaki et al., 2018b, Misaki et al., 2018a, Misaki et al., 2019, Misaki et al., 2021, Nicholson et al., 2016, Nicholson et al., 2018, Nicholson et al., 2021, Weaver et al., 2020, Zhao et al., 2023, Zotev et al., 2018, Zweerings et al., 2018, Zweerings et al., 2020). Further supporting its clinical potential, two recent meta-analyses report moderate-to-large reductions in PTSD symptoms following neurofeedback—including both EEG and fMRI approaches—with effects sustained at follow-up timepoints (Choi et al., 2023, Voigt; et al., 2024). It has been hypothesized that regulating key network hubs using fMRI-NFB could facilitate the restoration of alterations within large-scale intrinsic connectivity networks (ICNs), thereby helping to reduce associated symptoms (Koek et al., 2019, Lanius et al., 2015, Nicholson et al., 2020a, Nicholson et al., 2020b, Sheynin et al., 2020, Szeszko and Yehuda, 2019). Three core ICNs—the central executive network (CEN), salience network (SN), and default mode network (DMN)—are fundamental to understanding the neural basis of higher cognitive functions and transdiagnostic symptoms in psychiatric illnesses (Menon, 2011, Menon, 2020). Notably, alterations in these networks have been strongly implicated in the pathophysiology of PTSD (e.g., Fenster et al., 2018, Lanius et al., 2015, Nicholson et al., 2024; Harricharan et al., 2020, Shalev et al., 2024, Shin and Liberzon, 2010).

The CEN, a frontoparietal and cerebellar network centered on the dorsolateral prefrontal cortex (dlPFC), is critical for cognitive control processes such as emotion regulation, working memory, and behavioral modulation (Akiki et al., 2017, Etkin et al., 2015, Habas et al., 2009, Koechlin and Summerfield, 2007; Miller and Cohen, 2001, Petrides, 2005, Seeley et al., 2007). Dysfunction of the CEN, particularly dlPFC hypoactivity, is a transdiagnostic feature of PTSD, anxiety and mood disorders, emphasizing the importance of effective CEN recruitment for emotion regulation (for review, Zilverstand et al., 2017). The SN, with key nodes in the amygdala, insula and dorsal anterior cingulate cortex (dACC), facilitates the detection and prioritization of emotionally salient stimuli, interoceptive processing, and autonomic regulation (Dosenbach et al., 2007, Gogolla, 2017, Modinos et al., 2009, Namkung et al., 2017, Seeley et al., 2007, Sridharan et al., 2008). In PTSD, emotion dysregulation has been attributed to diminished top-down inhibitory control by the dlPFC and other CEN components over hyperactive SN regions, particularly the amygdala (Admon et al., 2013, Aupperle et al., 2012, Lanius et al., 2010, Pitman et al., 2012, Rauch et al., 2006, Ronzoni et al., 2016, Shin and Liberzon, 2010).

Consistent with this model, fMRI-NFB studies in PTSD have often focused on training participants to either enhance activation in frontal regions involved in emotion regulation (Zweerings et al., 2018, Zweerings et al., 2020) or reduce amygdala hyperactivity during negative emotion induction (Gerin et al., 2016, Nicholson et al., 2016, Nicholson et al., 2018, Zhao et al., 2023). Studies targeting amygdala downregulation have demonstrated increased dlPFC and vlPFC activation and broader recruitment of the CEN during emotion induction paradigms (Nicholson et al., 2016, Nicholson et al., 2018). Moreover, in another study, three sessions of amygdala-targeted fMRI-NFB were shown to reduce PTSD symptom severity, with improvements correlating with enhanced functional connectivity between the amygdala and dlPFC (Zotev et al., 2018). In the same dataset, a connectome-wide analysis revealed that increased resting-state connectivity between the left dlPFC and precuneus was associated with reductions in hyperarousal symptoms (Misaki et al., 2018a). Nevertheless, the fronto-limbic model alone does not fully account for the diverse symptoms observed in PTSD or widespread dysregulation observed across other large-scale brain networks (Akiki et al., 2017, Fenster et al., 2018, Kamiya and Abe, 2020).

The DMN, which comprises several midline brain regions including the posterior cingulate cortex (PCC), precuneus, and the medial prefrontal cortex (mPFC) (Andrews-Hanna et al., 2010, Buckner et al., 2008, Greicius et al., 2003; Qin and Northoff, 2011, Spreng et al., 2009), has recently garnered significant attention in PTSD due to its involvement in self-related processing, autobiographical memory, and social cognition—functions that are frequently perturbed in individuals with PTSD (Akiki et al., 2017, Bluhm et al., 2009, Daniels et al., 2010, Fenster et al., 2018, Frewen et al., 2020, Hinojosa et al., 2019, Kearney and Lanius, 2024, Lanius et al., 2015, Thome et al., 2020). Altered DMN connectivity has been reported in both resting-state and task-dependent contexts, including disrupted connectivity between anterior nodes (e.g., mPFC), posterior nodes (e.g., PCC, precuneus), and subcortical structures (e.g., hippocampus, thalamus) (Bluhm et al., 2009, Koch et al., 2016; D. R. Miller et al., 2017; Qin et al., 2012, Sripada et al., 2012). Graph-theoretical studies reveal increased connectivity within the posterior community of the DMN (involving the bilateral PCC and precuneus), relative to decreased connectivity within the anterior community of the DMN (involving the mPFC) during rest in PTSD (Akiki et al., 2018, Holmes et al., 2018, Kennis et al., 2016, Shang et al., 2014). During executive functioning tasks, individuals with PTSD show maladaptive increases in intra-DMN connectivity rather than increases in DMN connectivity with SN and CEN regions as shown by healthy controls (Daniels et al., 2010). Meta-analyses have further highlighted PCC hyperactivity among individuals with PTSD compared to healthy controls during trauma-related autobiographical memory retrieval (Thome et al., 2020) and traumatic imagery tasks (Ramage et al., 2013). Taken together, these findings highlight the PCC as a potential target for neurofeedback interventions aimed at alleviating PTSD symptoms.

fMRI-NFB has previously been used to downregulate PCC activity during a trauma/emotion provocation paradigm among PTSD participants and healthy controls (Lieberman et al., 2023a, Lieberman et al., 2023b, Nicholson et al., 2021). Both participant groups successfully downregulated PCC activity; however, unique within-group decreases in activation were observed in the PTSD group across several brain regions, including key nodes of the DMN (bilateral dmPFC, hippocampus) and SN (amygdala, mid-cingulate cortex) during neurofeedback training (Nicholson et al., 2021). The PTSD group also displayed distinct functional connectivity patterns between the PCC and other regions, including the dmPFC and vmPFC within the DMN, as well as the amygdala and insula within the SN. These connectivity patterns were associated with baseline clinical measures such as PTSD symptom severity, depression, difficulties in emotion regulation, childhood trauma, and dissociation, suggesting a potential recalibration of disrupted ICN dynamics (Lieberman et al., 2023a). Interestingly, only the healthy control group exhibited increased activation in a CEN region—the right dlPFC. Notably, right dlPFC activation during neurofeedback training was negatively correlated with baseline PTSD symptom severity scores and difficulties in emotion regulation, further suggesting that the CEN may be involved in PCC regulation for healthy individuals (Nicholson et al., 2021). In a direct comparison of PCC- and amygdala-targeted fMRI-NFB studies, PCC downregulation was uniquely associated with reductions in reliving and distress symptoms, accompanied by concomitant decreases in PTSD-related brain activity, including within the cuneus/precuneus/primary visual cortex, the superior parietal lobule, the occipital pole, and the superior temporal gyrus/temporoparietal junction (Lieberman et al., 2023b). Taken together, these findings suggest a divergence in the neural mechanisms underlying different fMRI-NFB targets in PTSD. While amygdala-targeted neurofeedback relies heavily on dlPFC involvement and broader CEN recruitment to exert top-down control over hyperactive limbic regions, PCC-targeted neurofeedback appears to predominantly engage DMN-centred mechanisms. This distinction aligns with emerging evidence that the DMN plays a central role in facilitating emotion regulation (e.g., Fresco et al., 2017, Messina et al., 2021).

While the CEN’s role in emotion regulation is well established, research increasingly highlights the DMN’s contributions to this process (Fresco et al., 2017, Garrett et al., 2019, Lanius et al., 2010, Messina et al., 2021, Sheynin et al., 2020, Zhou et al., 2012). For instance, regulation of the PCC has been linked to emotional acceptance (Messina et al., 2021), mindfulness mediation (Garrison et al., 2013a, Garrison et al., 2013b), and reductions in PTSD symptoms following trauma-focused cognitive behavioural therapy (Garrett et al., 2019). These findings suggest that modulating PCC hyperactivity in response to trauma provocation may represent an important avenue toward therapeutic response. However, it remains uncertain whether PCC downregulation is predominantly mediated by inter-network regulation via the CEN, as observed during amygdala-targeted neurofeedback, or if it relies more heavily on intra-network mechanisms within the DMN, particularly in a PTSD context. Addressing this question requires a network-based approach capable of simultaneously characterizing DMN and CEN dynamics, providing critical insight into the mechanisms underlying emotion regulation during PCC-targeted fMRI-NFB in PTSD.

### Current study

In the current study, we investigated the differential contributions of DMN and CEN neural mechanisms in facilitating emotion regulation during PCC downregulation via fMRI-NFB among individuals with PTSD and healthy controls. Following a network-based approach, we applied independent component analysis (ICA) to examine neurofeedback-mediated changes in DMN and CEN functional connectivity during a trauma/emotion provocation paradigm. During this paradigm, participants viewed personalized trauma-related (PTSD group) or distressing (healthy controls) words while undergoing a single session of fMRI-NFB. We used ICA to decompose fMRI data into independent spatial components and associated time courses. The resultant subject-specific spatial maps quantified the degree to which each voxel was functionally integrated with a given network, providing a measure of network-to-voxel association strength (i.e., network functional connectivity). Comparing these maps across groups and conditions thus enabled inferences about differences in how each network is functionally expressed and coupled with other brain regions. We hypothesized that PCC downregulation during fMRI-NFB would primarily involve increases in DMN connectivity, reflecting intra-network mechanisms that address self-referential and emotional dysregulation, both of which are characteristic of PTSD. Emerging evidence highlights the DMN’s critical role in facilitating emotion regulation, suggesting that DMN-mediated processes may be central to the mechanisms underlying PCC downregulation among individuals with PTSD.

## 2 Methods

### 2.1 Participants

The sample for this study consisted of 30 participants, including 15 individuals diagnosed with PTSD and 15 healthy controls. One participant in the PTSD group was excluded from the analysis due to self-reportedly falling asleep during the transfer run, leaving a final sample of 14 individuals in the PTSD group and 15 in the healthy control group (Table 1). The sample size was based on study feasibility during the recruitment period. There were no significant differences between the groups in terms of biological sex or handedness. However, the mean age of participants in the PTSD group was significantly higher than that of the healthy control group. Importantly, when age was included as a covariate within the analyses, no significant effect was found for age or its interaction with group. No participants reported a significant history of head injuries.

**Table 1:** Demographic and clinical information.

	**PTSD Group (N = 14)**	**Healthy Control Group (N = 15)**
Biological Sex	6F, 8 M	10F, 5 M
Years of Age*	49.5 (±5.1)	37.7 (±12.9)
CAPS-5	43.2 (±8.3)	0 (±0)
BDI	32.1 (±12.6)	1.2 (±2.5)
CTQ	61.5 (±25.8)	31.1 (±8.4)
MDI	87.4 (±28.2)	43.2 (±4.4)
DERS	107.6 (±24.8)	52.8 (±9.0)
Psychotropic Medication	10	0
MDD − Current	9	0
MDD − Past	2	0
Other Psychiatric Conditions − Current	5	0

Values in parentheses indicate standard deviations. Other psychiatric conditions in the PTSD group included agoraphobia (N = 1), panic disorder (N = 1), and somatic symptom disorder (N = 3). *The mean age was significantly higher in the PTSD group compared to the healthy control group. Abbreviations: CAPS-5 = Clinician-Administered PTSD Scale-5; BDI = Beck's Depression Inventory; CTQ = Childhood Trauma Questionnaire (*none or minimal childhood trauma* = 25–36, *moderate* = 56–68, *extreme trauma* > 72); MDI = Multiscale Dissociation Inventory; DERS = Difficulties in Emotion Regulation Scale; MDD = Major Depressive Disorder.

Participants were recruited between 2017 and 2019 via clinician referrals, community programs for traumatic stress, and posters within the London, Ontario community. Inclusion criteria for the PTSD group included a current primary diagnosis of PTSD as measured by the Clinician-Administered PTSD Scale (CAPS-5) (Weathers et al., 2018) and the Structured Clinical Interview for DSM-5 (SCID) (First, 2015). Participants in the PTSD group who were currently taking psychotropic medication were required to be on a stable dose for at least one month prior to participating in the study. Exclusion criteria for the PTSD group included ongoing or recent (within the previous three months) alcohol or substance use disorders, suicidal ideations, self-injurious behaviours requiring medical attention, and lifetime diagnoses of bipolar or psychotic disorders. Exclusion criteria for the control group included a lifetime diagnosis of psychiatric illness and current use of psychotropic medications. Exclusion criteria for all participants included previous biofeedback treatment, noncompliance with 3 T fMRI safety guidelines, untreated medical conditions, pregnancy, previous head injury with loss of consciousness, and neurological or pervasive developmental disorders. The study was approved by the Research Ethics Board at Western University, and all participants provided written informed consent and received financial compensation for their participation. A CRED-nf checklist (Ros et al., 2020) summarizing experimental design was completed using the standardized online tool (https://crednf.shinyapps.io/CREDnf/) and is included in the supplementary material.

Prior to scanning, all participants completed several clinical assessments, including the Beck’s Depression Inventory (BDI) (Beck et al., 1997), the Childhood Trauma Questionnaire (CTQ) (Bernstein et al., 2003), Difficulties in Emotion Regulation Scale (DERS) (Gratz & Roemer, 2003), and the Multiscale Dissociation Inventory (MDI) (Briere et al., 2005). After each of the fMRI neurofeedback runs, participants completed the Response to Script Driven Imagery Scale (RSDI) (Hopper et al., 2007), which included the following symptom subscales: reliving, distress, physical reactions, dissociation, and emotional numbing.

### 2.2 Real-time fMRI neurofeedback protocol

**Fig. 1:**
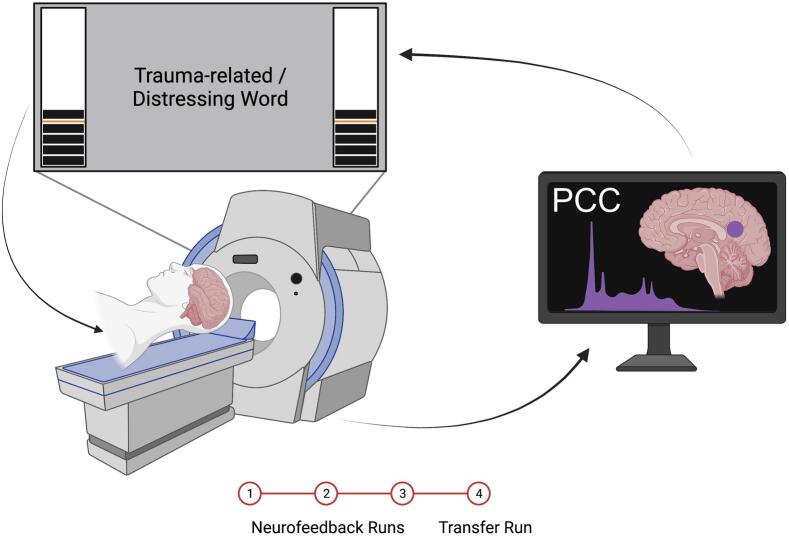
Illustration of the fMRI-NFB experimental set-up. The neurofeedback signal took the form of a virtual thermometer whose level changed in response to fluctuating activity within the neurofeedback target region (PCC). Participants viewed the neurofeedback signal during a trauma/emotion provocation paradigm while they were in the scanner. All participants completed three neurofeedback training runs, followed by a transfer run, in which they were not shown the neurofeedback signal. Figure

In this study, we used an identical experimental protocol and neurofeedback paradigm as described in our previous publications (Lieberman et al., 2023a, Lieberman et al., 2023b, Nicholson et al., 2018, Nicholson et al., 2021; Nicholson, Rabellino, et al., 2016) (Fig. 1). During neurofeedback training, participants were shown a signal indicating PCC activation. This signal was presented as two thermometers on either side of a screen visible to participants inside the scanner. The bars on the thermometers increased or decreased based on changes in the PCC's BOLD signal. Each segment in the thermometer represented a 0.2 % change in PCC activation, with a maximum range of 2.8 % increase and 1.2 % decrease from baseline. At the start of each trial, the mean of the preceding four data points was taken as the baseline and displayed to participants as an orange line on the thermometer. Participants were not provided with specific regulation instructions but were told that they would be “regulating an area of the brain related to emotional experience.” They were instructed to focus on the presented word for the duration of each condition, while using their peripheral vision to monitor the thermometers. Before the first run, participants were informed of the delay (∼6–8 s) in the neurofeedback signal due to the BOLD signal time lag and real-time processing.

reproduced with permission from Nicholson et al. (2021).

Our neurofeedback protocol consisted of three conditions: regulate, view, and neutral. In the regulate condition, participants were instructed to decrease the neurofeedback signal while viewing a personalized trauma-related word (PTSD group) or a matched distressing word (control group). In the view condition, participants viewed the chosen trauma/distressing words but were instructed to respond naturally and not attempt to exert regulatory control over the neurofeedback signal. In the neutral condition, participants viewed a personalized neutral word and were instructed to respond naturally and not attempt to exert regulatory control over the neurofeedback signal. Personalized words were selected by each participant with the guidance of a trauma-informed clinician (10 trauma/distressing and 10 neutral words). For participants in the PTSD group, the words were directly related to their individual trauma experiences; for controls, they were drawn from their most stressful life events. To match emotional salience across participants, each individual was instructed to select words that they personally rated as approximately 7 out of 10 in terms of subjective units of distress. This within-subject calibration ensured that stimuli elicited a consistent moderate emotional response while avoiding excessive distress, in keeping with safety and well-being considerations for participants. Stimuli were presented using Presentation software from Neurobehavioral Systems.

Participants completed three consecutive neurofeedback training runs, followed by one transfer run. The transfer run was identical to the training runs except that participants were not shown any neurofeedback signal. Each run lasted 9 min and included 15 trials (5 per condition). The timing for all trials was as follows: 2 s for instructions, followed by 24 s for the condition, and finally a 10-second implicit resting state with an intertrial fixation cross. The condition order was counterbalanced for all trials.

### 2.3 Real-time signal processing for neurofeedback

To present real-time PCC neural activation to participants via a thermometer display, we first imported anatomical scans into BrainVoyager (version QX2.4, Brain Innovations), skull-stripped and transformed them into Talairach space. We then added the normalization parameters into TurboBrainVoyager (TBV, version 3.0, Brain Innovations). TBV was the software used for real-time signal processing and analysis of BOLD signals. During real-time processing, TBV detected and corrected for head movements (using a rigid body transformation) and conducted spatial smoothing using a 4-mm full-width-half-maximum (FWHM) Gaussian kernel. The first two functional scan volumes were removed prior to real-time signal processing.

The neurofeedback target, the PCC, was defined using a 6 mm sphere at the coordinate (MNI: 0, –50, 20) (Bluhm et al., 2009). We used the “best voxel selection” tool in TBV to calculate the BOLD signal amplitude in this target area. This method identifies the 33% most active voxels (i.e., the voxels with the highest beta-values) for the view > neutral contrast. The first two trials of each neurofeedback run were the view and neutral conditions which allowed us to select voxels based on the view > neutral contrast. As outlined in previous publications (Lieberman et al., 2023a, Lieberman et al., 2023b; Nicholson et al., 2016, Nicholson et al., 2018; Paret et al., 2014, Paret et al., 2016), the voxel selection was dynamically updated throughout the duration of training based on (a) the voxel with the largest beta value, and (b) the magnitude of deviation from the mean of all condition betas (Goebel, 2014). This method eliminates inter-subject differences in the number of voxels used for signal extraction while accounting for slight anatomical shifts across runs and/or movement-related slice shifts. In order to create a smoother neurofeedback signal, the mean of the neurofeedback signal of the current and three preceding TRs was shown to participants (via thermometer display) (Lieberman et al., 2023a, Lieberman et al., 2023b; Nicholson et al., 2016, Nicholson et al., 2018; Paret et al., 2014, Paret et al., 2016).

### 2.4 fMRI image data acquisition and preprocessing

In this study, we used a 3 Tesla MRI Scanner (Siemens Biograph mMR) at the Lawson Research Institute with a 32-channel head coil. Foam padding was used to stabilize participants' heads during scanning. We acquired functional whole-brain images of BOLD contrasts using a gradient echo T2*-weighted echo-planar-imaging sequence (TE = 30 ms, TR = 2 s, FOV = 192x192mm, flip angle = 80°, in-plane resolution = 3x3mm). One volume consisted of 36 ascending interleaved slices tilted −20° from the AC-PC orientation. Volumes had a thickness of 3 mm and a slice gap of 1 mm. The experimental runs comprised 284 volumes each, and T1-weighted anatomical images were obtained using a Magnetization Prepared Rapid Acquisition Gradient Echo sequence (TE = 3.03 ms, TR = 2.3 s, 192 slices, FOV = 256x256mm).

We preprocessed functional images using SMP12 within MATLAB R2020a. We followed our standard preprocessing routine, which included discarding the first four volumes, slice time correction to the middle slice, reorientation to the AC-PC axis, spatial alignment to the mean image via rigid body transformation, reslicing, and coregistration of the functional mean image to the participant's anatomical image. We visually inspected the co-registration for all participants and performed corrections, if necessary. We segmented the co-registered images using the “New Segment” method in SPM12. After this, we normalized the functional images to the MNI standard template and smoothed them using a 6-mm FWHM Gaussian kernel. Finally, we conducted additional motion correction using the Artifact Detection Tool (ART) software package (https://www.nitrc.org/projects/artifact_detect), which computes regressors to account for outlier volumes and movement.

### 2.5 Statistical analysis

This study presents a secondary analysis of imaging data previously reported in prior publications (Lieberman et al., 2023a, Lieberman et al., 2023b, Nicholson et al., 2021). Nicholson et al. (2021) presented the original study findings, including PCC downregulation success, activation-based analyses, clinical correlations, and a machine learning classification analysis. Lieberman et al. (2023a) examined task-based PCC functional connectivity using psychophysiological interaction (PPI) analyses, while Lieberman et al. (2023b) compared outcomes in the PTSD group to those from a prior amygdala-targeted fMRI-NFB study (Nicholson et al., 2016, Nicholson et al., 2018) to examine target-specific neural and clinical effects. The current analysis introduces a novel network-based approach using ICA to investigate DMN and CEN dynamics during PCC downregulation.

#### 2.5.1 Independent component analysis

Independent component analysis (ICA) is a computational technique that identifies statistically independent sources from multivariate data, making it an effective approach for analyzing functional connectivity within large-scale brain networks (Beckmann, 2012). Unlike seed-based functional connectivity analyses, which focus on pre-defined regions of interest, ICA uses a data-driven approach to extract independent spatial maps (SMs) and associated time courses by considering relationships between all voxels (Calhoun et al., 2001, Calhoun et al., 2009). In this study, we applied ICA to identify data-driven representations of the DMN and CEN and to examine neurofeedback-mediated changes in their functional connectivity during PCC downregulation in individuals with PTSD and healthy controls.

To determine the optimal number of independent components (ICs) for this analysis, we used the GroupICA of fMRI Toolbox (GIFT) with the Infomax algorithm and minimum description length (MDL) criteria, which yielded 37 components. This step was performed in GIFT because the CONN toolbox (Whitfield-Gabrieli & Nieto-Castanon, 2012) does not include a function for estimating the optimal number of ICs in a data-driven manner. Following this, the ICA itself was conducted in CONN (version 21a), where group-level analysis was performed on concatenated functional imaging data from all participants (n = 29) and study conditions (including rest, instructions, neurofeedback training, and transfer run time blocks). The analysis in CONN began with dimensionality reduction to retain the dominant variance components and improve the stability of subsequent ICA estimation. Independent spatial components were then estimated using the FastICA algorithm, and the resulting group-level components were back-reconstructed into single-participant SMs using the GICA3 algorithm (Erhardt et al., 2011). A data-driven ICA approach was selected to account for potential differences in ICNs between PTSD participants and healthy controls, as prior research suggests these networks differ significantly between the two groups (Daniels et al., 2010, Nicholson et al., 2018; Harricharan et al., 2020, Rabellino et al., 2015, Shang et al., 2014, Tursich et al., 2015).

#### 2.5.2 First-level design matrix

Each neurofeedback training run and the transfer run were modeled as separate sessions. Within each session, task events—including the initial rest period, instructions, and study conditions—were modeled as block regressors convolved with the hemodynamic response function (HRF). Functional data was subjected to high-pass temporal filtering to remove low-frequency drifts and serial correlations were addressed using an autoregressive AR(1) model. On the single-subject level, the back-reconstructed component time courses were regressed onto the HRF-convolved task design matrix to estimate condition-specific β-weights for each network. The resulting β-maps for each subject were then carried forward for subsequent second-level analyses.

#### 2.5.3 Network connectivity analysis and clinical correlations

To assess group differences in the SMs during neurofeedback training, we conducted separate mixed-design two (Group: PTSD, healthy control) x three (Run: 1, 2, 3) x two (Condition: regulate, view) repeated measures ANOVAs for each selected component. These analyses were restricted to the three neurofeedback training runs and did not include the transfer run, as we were primarily interested in examining connectivity changes directly associated with active neurofeedback-driven regulation. For both the DMN and CEN components, all main effects and interaction terms were modeled and tested. Based on our hypothesis regarding network-level mechanisms of PCC-targeted neurofeedback, we conducted a priori planned within- and between-group comparisons of regulate versus view conditions (averaged across all three training runs). Additionally, we evaluated changes in network functional connectivity over the course of neurofeedback training by comparing regulate and view conditions during run three versus run one, and vice versa. Subsequently, we conducted an exploratory analysis to directly compare the functional connectivity of the DMN versus the CEN during neurofeedback training. To do so, we utilized a mixed-design two (Component: DMN, CEN) x two (Group: PTSD, healthy control) x three (Run: 1, 2, 3) x two (Condition: regulate, view) repeated measures ANOVA, modelling only the component by condition interaction at each level of group, across neurofeedback training runs. We focused specifically on the contrast of regulate versus view conditions between networks (i.e., [DMN_regulate vs DMN_view] vs [CEN_regulate vs CEN_view]), allowing us to assess differences in neurofeedback-related modulation between the two networks for each level of group.

Additionally, we conducted linear regression analyses across all subjects to assess correlations between clinical measures and network functional connectivity during neurofeedback training. First, for each subject, the ImCalc function in SPM was used to create separate concatenated regulate and view SMs across the three neurofeedback training runs. These concatenated regulate and view SMs were then imported back into ImCalc to create subtraction (regulate > view) SMs for each subject. Finally, these subtraction SMs were correlated with participant scores for CAPS-5 total, DERS, CTQ, BDI, MDI, and RSDI. All statistical tests were corrected for multiple comparisons using a cluster-level false discovery rate (FDR) significance threshold of *p* < 0.05, *k* = 10, with an initial cluster defining threshold of *p* < 0.001, *k* = 10 (Chumbley and Friston, 2009, Genovese et al., 2002, Kessler et al., 2017, Roiser et al., 2016). All ANOVAs, a priori planned contrasts, and linear regression analyses were conducted in SPM12.

## 3 Results

### 3.1 PCC downregulation with neurofeedback

**Fig. 2:**
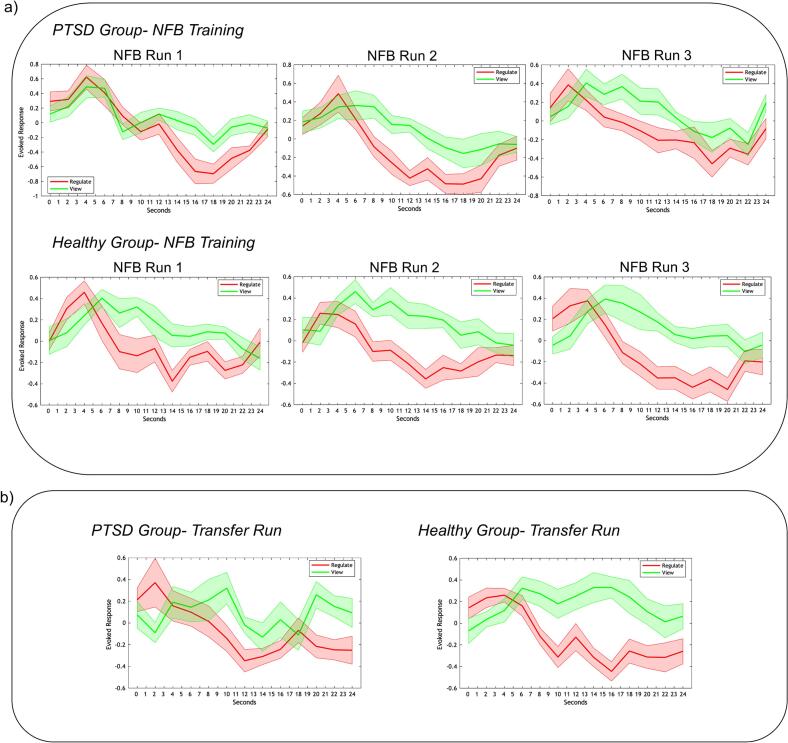
Event-related BOLD response in the PCC (NFB target region) during regulate and view. PTSD and healthy control groups during (a) the three NFB training runs and (b) the transfer run (no feedback provided). Red lines show the regulate condition (downregulation while viewing trauma/stressful words), and green lines show the view condition (passive viewing of trauma/stressful words). In both groups, PCC activation was significantly lower during regulate compared with view across all training runs and the transfer run. Groups showed comparable downregulation, with no significant differences in BOLD response between them in any run. The x-axis represents time across the 24-s conditions, and the y-axis indicates the event-related BOLD response (peristimulus time histogram). Shaded areas represent the standard error of the mean. (For interpretation of the references to colour in this figure legend, the reader is referred to the web version of this article.)

Reproduced with permission from Nicholson et al. (2021).

Both the PTSD and healthy control groups successfully downregulated PCC activity during regulate compared with view conditions. Detailed statistical analyses of these effects, including repeated measures condition-by-run ANOVAs and between-group comparisons for each condition are reported in Nicholson et al. (2021); here, we provide a brief summary for context. For both groups, the average event-related BOLD response within the PCC (NFB target region) was significantly lower during regulate than view for each of the three NFB training runs (Fig. 2a) as well as the transfer run (Fig. 2b). No significant main effects of run or run-by-condition interactions were observed, indicating that PCC downregulation occurred to a similar extent across runs. Additionally, there were no significant differences in the BOLD response within the PCC between the PTSD and healthy control groups during the regulate and view conditions. This implies that both groups were able to successfully downregulate their PCC to a similar extent; however, as observed in prior analyses (Lieberman et al., 2023a; Nicholson et al., 2021) as well as current findings (Section 3.3 Network connectivity analysis), the neural mechanisms supporting this regulation differed substantially between groups. Methodological details on the event-related BOLD response extraction and calculation—using rfxplot software to generate peristimulus time histograms from the PCC target sphere via a Finite Impulse Response (FIR) model (Gläscher, 2009)—are provided in Nicholson et al. (2021). Additionally, event-related BOLD responses within the PCC NFB target for all three conditions (neutral, view, regulate) are provided in the Supplementary Materials (Fig. S1).

### 3.2 Spatial sorting analysis: Component identification

Following ICA decomposition, we identified and excluded 21 components as noise or artifacts based on visual inspection (e.g., components dominated by ventricles, white matter, edge effects, or motion-related signals). The remaining 16 non-noise components were visually inspected for overlap with canonical DMN and CEN architecture and were also spatially sorted using CONN templates to aid in network identification.

**Fig. 3:**
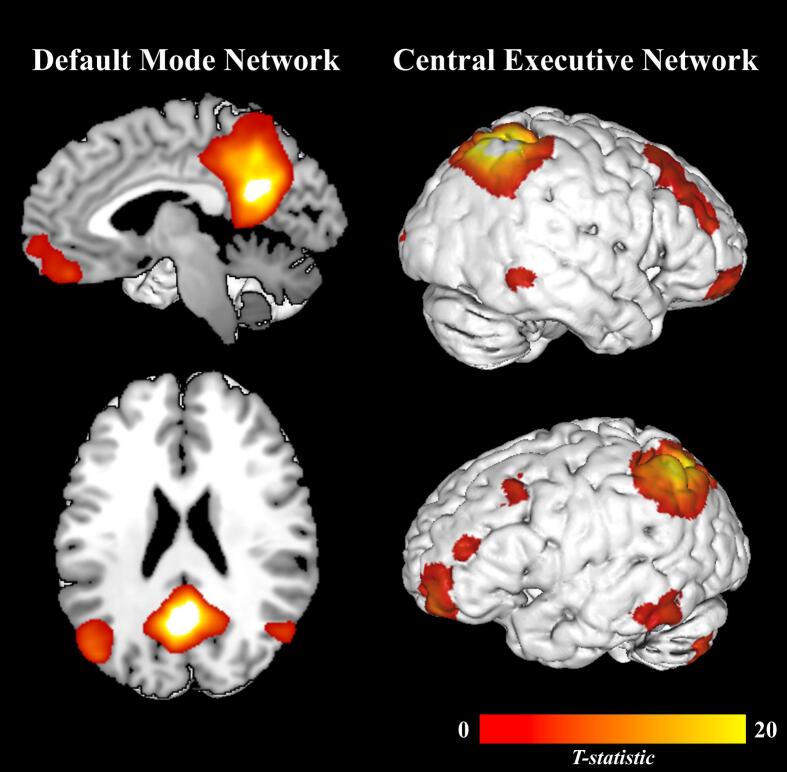
Spatial maps of the selected independent components. Brain maps depict the independent components representing the Default Mode Network (DMN; left) and Central Executive Network (CEN; right) derived from independent component analysis. The color scale indicates the *t* values for each independent component, averaged across all participants and experimental conditions, with results whole-brain FDR-corrected at *p* < 0.05, k = 10.

Of these, IC 1 and IC 5 were the only components that plausibly reflected the DMN and CEN, respectively, based on both visual inspection and spatial correspondence with CONN templates (Fig. 3). IC 1 showed a strong correlation with the DMN template (*r* = 0.54) and primarily encompassed the medial prefrontal cortex, bilateral angular gyri, posterior cingulate cortex/precuneus, and, to a lesser extent, the bilateral hippocampus. IC 5 demonstrated a moderate correlation with the frontoparietal network template (*r* = 0.39) and included the bilateral superior and middle frontal gyri (including the dlPFC), bilateral frontal poles, bilateral posterior parietal cortices, and, to a lesser extent, portions of the bilateral superior temporal gyri and the left cerebellum. Notably, the order of components here does not reflect coherence or explained variance (as in PCA); rather, component numbering is arbitrary and determined by the algorithm’s internal stochastic processes (Du and Fan, 2013, Himberg et al., 2004, Svensén et al., 2002). We proceeded with ICs 1 and 5 (hereafter referred to as the DMN and CEN, respectively) for all subsequent analyses. All other non-noise components are presented in the supplementary materials (Fig. S2) to provide full transparency regarding the ICA decomposition and component selection process.

### 3.3 Network connectivity analysis

F-test results for the repeated measures ANOVA for each component are shown in Table 2. The ANOVA for the DMN component revealed significant main effects of group and run. While no interaction terms reached statistical significance, the group by condition interaction yielded two clusters, the left precentral gyrus and right anterior insula (p = 0.052), which we report given their proximity to conventional significance thresholds and theoretical relevance. The ANOVA for the CEN component revealed significant main effects of group, run, and condition, but none of the interaction terms were statistically significant.

**Table 2:** Repeated measures ANOVA results for DMN and CEN functional connectivity.

				**MNI Coordinate**					
**Comparison**	**Brain region**	**H**	**k**	**x**	**y**	**z**	**F-stat.**	**Z-score**	***p*-FDR**
**DMN**									
Main effect of group	Superior frontal gyrus	R	51	16	44	52	34.04	5.43	0.047
	Anterior cingulate cortex		187	−2	44	0	28.68	5.00	<.001
	Superior frontal gyrus	L	93	–22	20	44	25.68	4.74	0.013
	Precuneus/cuneus	R	227	18	−78	46	23.53	4.54	<.001
	Superior frontal gyrus	L	63	−10	40	50	22.50	4.44	0.037
	dmPFC		85	−4	62	24	20.58	4.24	0.014
	Precuneus/cuneus	L	54	−10	−76	38	17.78	3.94	0.047
	Cuneus	R	50	22	−66	16	16.16	3.75	0.047
Main effect of run	Superior occipital gyrus	R	167	26	−80	18	13.15	4.41	0.001
Group x Condition	*Precentral gyrus*	*L*	*70*	*−52*	*8*	*26*	*29.11*	*5.04*	*0.052*
	*Anterior insula*	*R*	*76*	*36*	*24*	*−4*	*24.69*	*4.65*	*0.052*
**CEN**									
Main effect of group	Angular gyrus	R	112	44	−52	60	23.62	4.54	0.012
	Postcentral gyrus	R	129	18	−38	76	22.85	4.47	0.012
	Anterior insula	R	75	46	16	−4	22.51	4.44	0.037
	Middle frontal gyrus	R	75	36	22	56	20.81	4.27	0.037
Main effect of run	Middle temporal gyrus	L	139	−58	−36	−10	15.74	4.87	0.003
	Angular gyrus	L	216	−46	−56	32	14.83	4.71	<.001
Main effect of condition	Superior parietal lobule	L	79	−36	−46	38	26.13	4.78	0.020
	Superior frontal gyrus	R	311	16	12	64	23.30	4.51	<.001
	Middle frontal gyrus	L	175	−46	26	34	21.07	4.29	0.001
	Supramarginal gyrus	L	194	−54	−44	42	21.05	4.29	<.001
	Supramarginal gyrus	R	341	58	−44	44	20.16	4.20	<.001
	Supplementary motor cortex		108	−6	−8	58	19.96	4.18	0.008
	Middle frontal gyrus	R	91	44	14	44	18.78	4.05	0.013
	Middle frontal gyrus	L	105	−48	40	8	16.32	3.77	0.008

Results of the mixed-design 2 (group) by 3 (run) by 2 (condition) repeated measures ANOVAs for DMN and CEN functional connectivity evaluated at the FDR-cluster corrected threshold for multiple comparisons (*p* < 0.05, k = 10). The comparison, brain region, hemisphere of the region (H), cluster size (k), MNI coordinates (x, y, z), F-Statistic (F-Stat.), Z-score, and significance (*p*-FDR) of each significant cluster are included as columns. Italicized rows indicate results with a trending statistical significance (*p* = 0.052). Abbreviations: R = Right; L = Left, dmPFC = dorsomedial prefrontal cortex.

#### 3.3.1 DMN

**Fig. 4:**
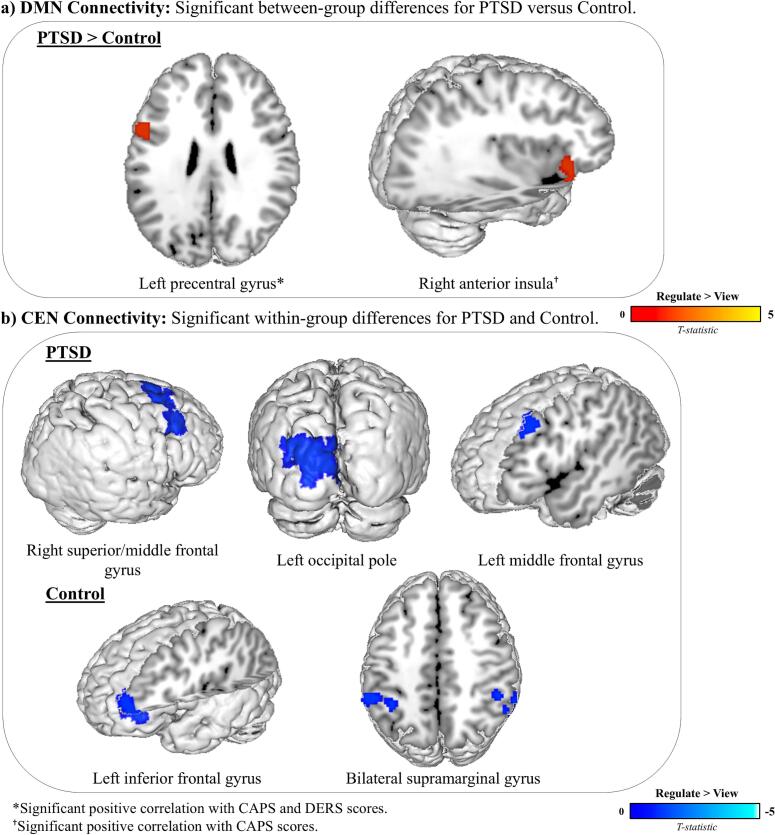
Significant connectivity differences for the DMN and CEN during PCC-targeted neurofeedback (regulate > view). (a) DMN connectivity: PTSD participants showed greater connectivity than controls with the left precentral gyrus and right anterior insula during regulate > view conditions. (b) CEN connectivity: PTSD participants showed decreased connectivity to the right superior frontal gyrus, left occipital pole, and bilateral middle frontal gyrus, while controls exhibited decreased connectivity to the left inferior frontal gyrus and bilateral supramarginal gyrus. Regions with increased connectivity during regulate > view are shown in red-yellow, and regions with decreased connectivity are shown in blue. Results are whole-brain FDR-corrected at *p* < 0.05, k = 10. Regions with connectivity that are significantly correlated with clinical measures are noted. (For interpretation of the references to colour in this figure legend, the reader is referred to the web version of this article.)

We observed significant between-group differences in DMN functional connectivity (Table 3). Specifically, for the PTSD compared to the control group, there was significantly increased connectivity between the DMN and the left precentral gyrus and right anterior insula during regulate compared to view conditions, averaged across all three neurofeedback training runs (Fig. 4a). Conversely, for both the PTSD and control groups, there were no significant within-group differences in DMN connectivity during regulate compared to view conditions and vice versa, averaged across the neurofeedback training runs. Similarly, for both participant groups, there were no significant within-group changes in DMN connectivity during regulate compared to view conditions, over the course of the neurofeedback training runs (i.e., run three versus run one, and vice versa).

**Table 3:** Within- and between-group differences in DMN functional connectivity.

					**MNI Coordinate**					
**Group**	**Condition**	**Brain region**	**H**	**k**	**x**	**y**	**z**	**t-Stat.**	**Z-score**	***p*-FDR**
*Within-group*										
PTSD	Reg > View	*ns*								
	View > Reg	*ns*								
Control	Reg > View	*ns*								
	View > Reg	*ns*								
*Between-group*										
PTSD > Control	Reg > View	Precentral gyrus	L	97	−52	8	26	5.40	5.17	0.044
		Anterior insula	R	102	36	24	−4	4.97	4.79	0.044
Control > PTSD	Reg > View	*ns*								

Results of the a priori within- and between-group comparisons of DMN functional connectivity for regulate versus view conditions averaged across all three neurofeedback training runs. Results were evaluated at the FDR-cluster corrected threshold for multiple comparisons (*p* < 0.05, k = 10). The group, condition, brain region, hemisphere of the region (H), cluster size (k), MNI coordinates (x, y, z), t-Statistic (t-Stat.), Z-score, and significance (*p*-FDR) of each significant cluster are included as columns. Abbreviations: *ns* = not significant; Reg = Regulate; R = Right; L = Left.

#### 3.3.2 CEN

**Fig. 5:**
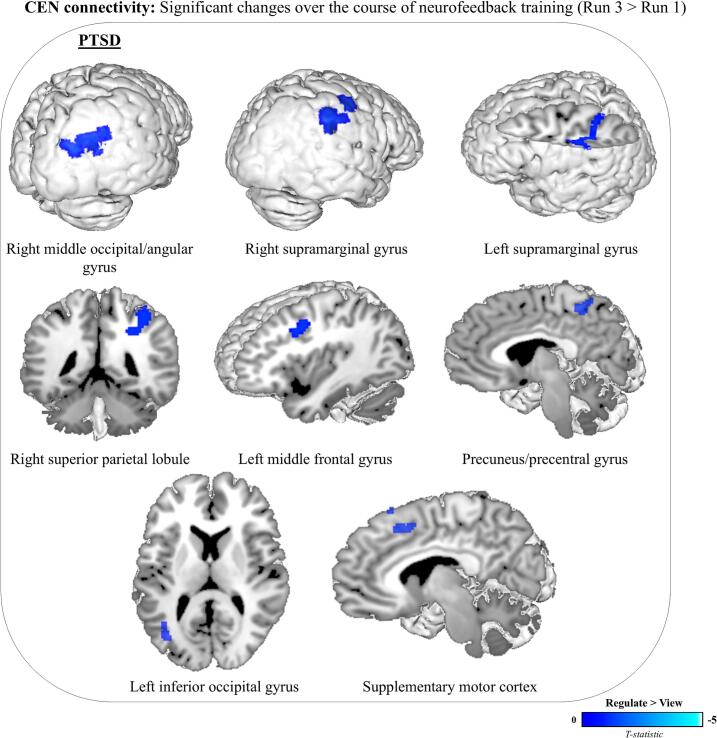
Progressive decreases in CEN connectivity over the course of neurofeedback training (run 3 > run 1). PTSD participants exhibited progressively decreased CEN connectivity during regulate > view conditions with several regions, including the right middle occipital/angular gyrus, bilateral supramarginal gyri, right superior parietal lobule, left middle frontal gyrus, precuneus/precentral gyrus, left inferior occipital gyrus, and supplementary motor cortex. No significant connectivity changes were observed for the control group. Decreased connectivity is shown in blue, with no regions exhibiting increased connectivity. Results are whole-brain FDR-corrected at *p* < 0.05, k = 10. (For interpretation of the references to colour in this figure legend, the reader is referred to the web version of this article.)

In contrast to the DMN, there were no significant between-group differences in CEN functional connectivity. However, for both the PTSD and healthy control groups, there were multiple significant within-group differences in CEN functional connectivity (Table 4). Specifically, the PTSD group showed significantly decreased functional connectivity between the CEN and the left occipital pole, the bilateral middle frontal gyrus, and the right superior frontal gyrus during regulate compared to view conditions, averaged across all three neurofeedback training runs (Fig. 4b). The control group exhibited significantly decreased functional connectivity between the CEN and the bilateral supramarginal gyrus and the left inferior frontal gyrus during regulate compared to view conditions, averaged across the training runs (Fig. 4b). For the PTSD group, there was progressively decreased CEN connectivity with several brain regions—the right middle occipital/angular gyrus, the right superior parietal lobule, the precuneus/precentral gyrus, the left inferior occipital gyrus, the bilateral supramarginal gyri, the supplementary motor cortex, and the left middle frontal gyrus—during regulate compared to view conditions, over the course of neurofeedback training (i.e., for run three compared to run one) (Fig. 5**,** Table 5). In contrast, no such progressive changes in CEN connectivity were observed for the control group during neurofeedback training.

**Table 4:** Within- and between-group differences in CEN functional connectivity.

					**MNI Coordinate**					
**Group**	**Condition**	**Brain region**	**H**	**k**	**x**	**y**	**z**	**t-Stat.**	**Z-score**	***p*-FDR**
*Within-group*										
PTSD	Reg > View	*ns*								
	View > Reg	Occipital pole	L	559	−12	−94	12	5.42	5.18	<.001
		Superior frontal gyrus	R	330	18	12	66	4.91	4.73	<.001
		Middle frontal gyrus	L	101	−52	20	40	4.55	4.41	0.037
		Middle frontal gyrus	R	102	42	14	48	3.98	3.89	0.037
Control	Reg > View	*ns*								
	View > Reg	Supramarginal gyrus	L	200	−36	−46	38	4.86	4.69	0.002
		Supramarginal gyrus	R	120	48	−42	52	4.06	3.95	0.017
		Inferior frontal gyrus	L	105	−50	42	6	4.02	3.92	0.021
*Between-group*										
PTSD > Control	Reg > View	*ns*								
Control > PTSD	Reg > View	*ns*								

Results of the a priori within- and between-group comparisons of CEN functional connectivity for regulate versus view conditions averaged across all three neurofeedback training runs. Results were evaluated at the FDR-cluster corrected threshold for multiple comparisons (*p* < 0.05, k = 10). The group, condition, brain region, hemisphere of the region (H), cluster size (k), MNI coordinates (x, y, z), t-Statistic (t-Stat.), Z-score, and significance (*p*-FDR) of each significant cluster are included as columns. Abbreviations: *ns* = not significant; Reg = Regulate; R = Right; L = Left.

**Table 5:** Progressive changes in CEN functional connectivity over the course of neurofeedback training.

						**MNI Coordinate**					
**Group**	**Condition**	**Run**	**Brain region**	**H**	**k**	**x**	**y**	**z**	**t-Stat.**	**Z-score**	***p*-FDR**
Control	Reg > View	3 > 1	*ns*								
	Reg > View	1 > 3	*ns*								
PTSD	Reg > View	3 > 1	*ns*								
	Reg > View	1 > 3	Middle occipital/angular gyrus	R	161	54	−70	26	4.68	4.52	0.015
			Superior parietal lobule	R	172	40	−40	48	4.55	4.40	0.015
			Precuneus/precentral gyrus		144	2	−38	52	4.52	4.38	0.020
			Inferior occipital gyrus	L	129	−36	−70	4	4.49	4.36	0.020
			Supramarginal gyrus	L	130	−54	−26	34	4.33	4.21	0.020
			Supplementary motor cortex		303	−2	2	54	4.23	4.12	0.001
			Supramarginal gyrus	R	132	62	−38	38	4.22	4.10	0.020
			Middle frontal gyrus	L	32	108	−36	10	4.13	4.02	0.040

Results of the within-group comparisons of CEN functional connectivity for regulate versus view conditions during neurofeedback training run three versus run one. Results were evaluated at the FDR-cluster corrected threshold for multiple comparisons (*p* < 0.05, k = 10). The group, condition, run, brain region, hemisphere of the region (H), cluster size (k), MNI coordinates (x, y, z), t-Statistic (t-Stat.), Z-score, and significance (*p*-FDR) of each significant cluster are included as columns. Abbreviations: *ns* = not significant; Reg = Regulate; R = Right; L = Left.

#### 3.3.3 DMN vs CEN

**Fig. 6:**
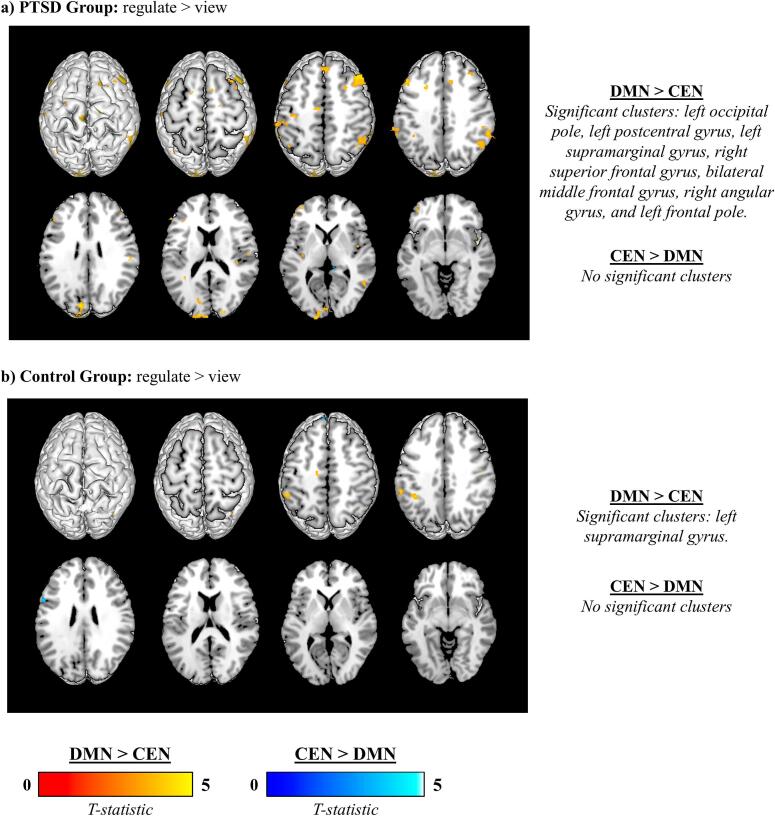
Differential functional connectivity of the DMN versus CEN during neurofeedback training (regulate > view). (a) PTSD group: The DMN exhibited significantly greater connectivity compared to the CEN with several regions, including the left occipital pole, left postcentral gyrus, left supramarginal gyrus, right superior frontal gyrus, bilateral middle frontal gyrus, right angular gyrus, and left frontal pole. (b) Control group: The DMN exhibited significantly greater connectivity compared to the CEN with the left supramarginal gyrus. For both groups, no regions showed significantly greater connectivity for the CEN compared to the DMN. For visualization purposes, all results surpassing the initial cluster-defining threshold (*p* < 0.001, k = 10) are displayed, but only clusters meeting the cluster-level FDR significance threshold (*p* < 0.05) are reported on the right side of the figure. Greater connectivity for DMN > CEN is shown in red-yellow, while greater connectivity for CEN > DMN is shown in blue. (For interpretation of the references to colour in this figure legend, the reader is referred to the web version of this article.)

The analysis which directly compared DMN versus CEN functional connectivity during regulate compared to view across all three neurofeedback training runs yielded several significant results (Table 6). Within the PTSD group, the DMN evidenced significantly increased connectivity to the left occipital pole, the left postcentral gyrus, the left supramarginal gyrus, the right superior frontal gyrus, the bilateral middle frontal gyrus, the right angular gyrus, and the left frontal pole, as compared to the CEN during regulate as compared to view (Fig. 6a). Within the control group, the DMN showed significantly increased connectivity to the left supramarginal gyrus, as compared to the CEN during regulate as compared to view (Fig. 6b). Conversely, for both the PTSD and control groups, the CEN did not yield any significant increases in connectivity, as compared to the DMN during regulate as compared to view. Taken together, these results suggest that the DMN exhibited greater task-related connectivity than the CEN during neurofeedback-specific modulation (i.e., Regulate > View). Importantly, this contrast captures task-related functional changes between the two networks, rather than differences in network architecture.

**Table 6:** Direct comparison of DMN versus CEN functional connectivity.

						**MNI Coordinate**					
**Group**	**Network**	**Condition**	**Brain region**	**H**	**k**	**x**	**y**	**z**	**t-Stat.**	**Z-score**	***p*-FDR**
PTSD	DMN > CEN	Reg > View	Occipital pole	L	460	−12	−96	12	5.11	5.01	<.001
			Postcentral gyrus	L	113	−56	−20	52	4.90	4.81	0.021
			Supramarginal gyrus	L	96	−48	−48	40	4.46	4.39	0.033
			Superior frontal gyrus	R	91	16	12	64	4.43	4.36	0.036
			Middle frontal gyrus	R	339	48	22	46	4.41	4.35	<.001
			Angular gyrus	R	365	50	−58	42	4.25	4.19	<.001
			Middle frontal gyrus	L	151	−46	22	36	4.20	4.14	0.007
			Frontal pole	L	125	−40	44	−2	4.13	4.07	0.016
			Supramarginal gyrus	L	97	−64	−38	40	3.94	3.89	0.033
	CEN > DMN	Reg > View	*ns*								
Control	DMN > CEN	Reg > View	Supramarginal gyrus	L	159	−36	−46	38	4.29	4.23	0.004
	CEN > DMN	Reg > View	*ns*								

Results of the exploratory within-group comparisons of DMN versus CEN functional connectivity for regulate versus view conditions averaged across all three neurofeedback training runs. Results were evaluated at the FDR-cluster corrected threshold for multiple comparisons (*p* < 0.05, k = 10). The group, network, condition, run, brain region, hemisphere of the region (H), cluster size (k), MNI coordinates (x, y, z), t-Statistic (t-Stat.), Z-score, and significance (*p*-FDR) of each significant cluster are included as columns. Abbreviations: DMN = Default Mode Network; CEN = Central Executive Network; Reg = Regulate; R = Right; L = Left; *ns* = not significant.

### 3.4 Clinical correlations

The linear regression analyses revealed significant correlations between DMN functional connectivity and baseline clinical measures during regulate compared to view conditions, averaged over neurofeedback training runs (Table 7 and Fig. 4a). Specifically, connectivity between the DMN and both the left precentral gyrus and right anterior insula showed positive correlations with participant total CAPS scores, indicating a relationship between DMN connectivity and PTSD symptom severity. Additionally, DMN connectivity with the left precentral gyrus was positively correlated with DERS scores, reflecting an association with difficulties in emotion regulation. No significant correlations were found between DMN connectivity and the other baseline clinical measures (CTQ, BDI, or MDI), or therapeutic outcomes (RSDI). Furthermore, CEN connectivity showed no significant associations with any clinical measures or treatment outcomes.

**Table 7:** Clinical correlations with network functional connectivity.

					**MNI Coordinate**					
**Clinical measure**	**Correlation**	**Brain region**	**H**	**k**	**x**	**y**	**z**	**t-Stat.**	**Z-score**	***p*-FDR**
*DMN*										
CAPS-5	+	Precentral gyrus	L	105	−50	8	26	6.50	5.00	0.029
	+	Anterior insula	R	101	36	26	−6	5.86	4.67	0.029
DERS	+	Precentral gyrus	L	109	−50	8	24	7.63	5.52	0.037

Results of the linear regression analyses between clinical scores and network functional connectivity averaged across all three neurofeedback training runs. Results were evaluated at the FDR-cluster corrected threshold for multiple comparisons (*p* < 0.05, k = 10). The clinical measure, correlation, brain region, hemisphere of the region (H), cluster size (k), MNI coordinates (x, y, z), t-Statistic (t-Stat.), Z-score, and significance (*p*-FDR) of each significant cluster are included as columns. Abbreviations: CAPS = Clinician-Administered PTSD Scale-5; DERS = Difficulties in Emotion Regulation Scale; R = Right; L = Left.

## 4 Discussion

In the present analysis, we applied an ICA—a data-driven, network-based approach—to examine the differential contributions of DMN and CEN neural mechanisms in facilitating emotion regulation during PCC-targeted fMRI-NFB among individuals with PTSD and healthy controls. Previous findings have shown that while both groups can successfully downregulate PCC activity, PTSD participants exhibit unique within-group decreases in neural activity across several regions, including key nodes of the DMN (i.e., bilateral dmPFC, hippocampus) and SN (i.e., amygdala, mid-cingulate cortex) during neurofeedback training (Nicholson et al., 2021). Additionally, PTSD participants display distinct patterns of functional connectivity between the PCC and other regions, including the dmPFC and vmPFC within the DMN and the amygdala and insula within the SN (Lieberman et al., 2023a). Comparative analyses of PCC- and amygdala-targeted fMRI-NFB have demonstrated that PCC downregulation is uniquely associated with reductions in reliving and distress symptoms, along with accompanying decreases in activity within PTSD-related brain regions (Lieberman et al., 2023b). Hence, unlike amygdala-targeted neurofeedback, which predominantly involved greater engagement of the CEN (Misaki et al., 2018a, Nicholson et al., 2016, Nicholson et al., 2018, Zotev et al., 2018), PCC-targeted neurofeedback appears to rely more heavily on DMN-centred mechanisms. Building on these findings, we used a network-based approach to directly examine whether PCC downregulation primarily involves intra-network DMN dynamics or regulation through inter-network interactions with the CEN.

Notably, the DMN component—which included the PCC ROI targeted during neurofeedback—showed increased connectivity with the precentral gyrus and anterior insula during regulate compared to view conditions for PTSD participants relative to healthy controls, averaged across all three neurofeedback training runs. These regions are implicated in PTSD psychopathology, and the observed connectivity increases were positively correlated with PTSD symptom severity and difficulties in emotion regulation, suggesting that neurofeedback may exert greater recalibration effects among individuals with more severe symptoms. Conversely, the CEN component showed decreased connectivity during regulate compared to view for both PTSD participants and healthy controls, averaged across the three neurofeedback training runs. Additionally, for the PTSD group, there was progressively reduced CEN connectivity over the course of neurofeedback training. Critically, direct comparisons revealed that DMN connectivity with several brain regions was consistently greater than CEN connectivity during PCC downregulation for both PTSD participants and healthy controls. Taken together, findings from this study suggest that PCC-targeted neurofeedback may primarily engage DMN-mediated processes, consistent with emerging neurobiological evidence highlighting the central role of the PCC and DMN in emotion regulation and autobiographical memory, particularly in the context of PTSD.

### 4.1 DMN connectivity

Our analysis revealed increased DMN connectivity with the left precentral gyrus and right anterior insula in PTSD participants compared to healthy controls during regulate versus view, highlighting potential DMN-specific mechanisms underlying PCC downregulation in the context of PTSD. The precentral gyrus, part of the sensorimotor network (SMN), is involved in processing and integrating sensory and motor information to facilitate bodily movement (Beckmann et al., 2005, Heba et al., 2017). In PTSD, disruptions in the neural representations of sensory and motor aspects of trauma-related autobiographical memories may contribute to alterations in bodily awareness and heightened somatic reactivity, potentially contributing to symptoms such as re-experiencing or reliving traumatic memories in the form of sensations and motoric actions (Brewin, 2001, Brewin et al., 1996, Kearney and Lanius, 2024, van Der Kolk and Fisler, 1995). Indeed, hypercoupling between the SMN and the posterior DMN may be a critical mechanism underlying flashback reliving experiences in PTSD (Kearney et al., 2023). Consistent with this, altered connectivity involving the pre- and post-central gyri has been reported in several studies of individuals with PTSD (Chen et al., 2019, Harricharan et al., 2020, Leroy et al., 2022, Rabellino et al., 2018; M. E. Shaw et al., 2002). Moreover, a recent meta-analysis of resting-state functional connectivity studies found the SMN to be hyperconnected to the DMN and affective networks in PTSD, with these connectivity results showing positive correlations with PTSD symptoms (Bao et al., 2021).

The anterior insula, a core node of the SN, is critically involved in processing interoceptive and exteroceptive sensory stimuli, attributing salience, and facilitating higher-order emotional and perceptual processes through interactions with frontal cortical regions (Beissner et al., 2013, Craig, 2009, Cauda et al., 2011, Uddin, 2015). Hyperactivity within the anterior insula is a well-documented marker of PTSD psychopathology and has been associated with hyperarousal, hyperreactivity to trauma-related stimuli, and disturbances in bodily self-consciousness (Akiki et al., 2017, Koch et al., 2016, McCurry et al., 2020; Harricharan et al., 2020, Rabinak et al., 2011, Sripada et al., 2012, Tursich et al., 2015, Yehuda et al., 2015). Under the triple-network model, the SN plays a pivotal role in switching between the DMN and CEN to coordinate responses to internally directed versus goal-oriented cognitive processes (Manoliu et al., 2014, Menon and Uddin, 2010, Uddin, 2015), a function that is often disrupted in PTSD (for review, Akiki et al., 2017). Notably, the right anterior insula has been shown to exert strong causal influence over nodes within the SN, CEN, and DMN, highlighting its critical role in coordinating large-scale network dynamics (Shaw et al., 2021, Sridharan et al., 2008).

Taken together, these findings suggest that PCC-targeted neurofeedback may recalibrate neural circuits related to emotion regulation, bodily awareness, and the ability to dynamically coordinate shifts between self-referential (DMN) and goal-directed (CEN) processes, functions that are often disrupted in PTSD. Importantly, both significant DMN connectivity results were correlated with clinical measures during regulate compared to view. Specifically, DMN-right anterior insula connectivity was positively correlated with CAPS scores, and DMN-left precentral gyrus connectivity was positively correlated with both CAPS and DERS scores. However, although changes in DMN connectivity were associated with baseline clinical measures, they did not correlate with changes in clinical outcomes (i.e., RSDI scores) following this single session of neurofeedback. Nevertheless, these correlation results suggest that neurofeedback may exert greater recalibration effects on these DMN connectivity patterns among individuals experiencing more severe PTSD symptoms. Notably, these findings align with emerging models suggesting that neurofeedback can act as a network-level intervention, inducing recalibration across large-scale brain networks even when targeting a single region (Krause et al., 2024). In line with this view, our results show that training the PCC—a key DMN hub—modulates broader network-level dynamics, including increased connectivity with salience and sensorimotor regions implicated in PTSD, particularly among individuals with greater symptom severity. This notion is further supported by convergent evidence from our previous seed-based functional connectivity analysis, wherein we observed increased connectivity between the PCC and regions of the anterior DMN (i.e., dmPFC, vmPFC) and SN (i.e., amygdala, insula) (Lieberman et al., 2023a). From a network neuroscience perspective, there is substantial evidence to suggest that the PCC may serve as a particularly effective neurofeedback target. It exhibits among the highest global brain connectivity values across both anatomical and functional connectivity measures (Buckner et al., 2009, Hagmann et al., 2008, Tomasi and Volkow, 2011) and plays a key role in maintaining homeostatic balance between large-scale brain networks to support diverse cognitive and affective functions (Leech et al., 2012, Leech and Smallwood, 2019). For a more in-depth discussion of the PCC as a neurofeedback target from this perspective, see Lieberman et al. (2023b).

### 4.2 CEN connectivity

In contrast to the DMN, both PTSD participants and healthy controls exhibited decreased CEN functional connectivity with several brain regions during regulate compared to view conditions, suggesting a more limited role for CEN connectivity in facilitating PCC downregulation. For the PTSD group, decreased CEN connectivity was observed with the left occipital pole, bilateral middle frontal gyrus, and right superior frontal gyrus, averaged across all three neurofeedback training runs. Over the course of neurofeedback training (i.e., run three compared to run one), PTSD participants also showed progressively reduced CEN connectivity to multiple regions, including the right middle occipital/angular gyrus, right superior parietal lobule, precuneus/precentral gyrus, left inferior occipital gyrus, bilateral supramarginal gyrus, supplementary motor cortex, and left middle frontal gyrus. In the control group, decreased CEN connectivity was observed with the bilateral supramarginal gyrus and the left inferior frontal gyrus during regulate compared to view conditions, averaged across the three training runs. Unlike the PTSD group, the control group did not exhibit progressively reduced CEN connectivity across neurofeedback runs.

These reductions in CEN connectivity may reflect a diminished engagement of cortical top-down emotion regulation processes from the CEN, whereby PCC downregulation may prioritize emotion regulation mechanisms within the DMN. While this pattern may appear to contradict the established role of lateral prefrontal regions in emotion regulation (Morawetz and Basten, 2024, Zhang et al., 2025), we believe it can be understood in the context of our broader findings. This interpretation aligns with the DMN’s established role in self-related processing and emotional regulation, and suggests that PCC downregulation relies more heavily on intra-network dynamics within the DMN than on inter-network contributions from the CEN. This pattern contrasts with findings from previous amygdala-targeted neurofeedback studies, which have demonstrated increased dlPFC activity, enhanced dlPFC connectivity with the amygdala, and broader recruitment of the CEN during neurofeedback training, as well as stable DMN recruitment (Nicholson et al., 2016, Nicholson et al., 2018, Misaki et al., 2018a, Zotev et al., 2018). Moreover, in one case these CEN-driven mechanisms co-occurred with reductions in PTSD symptomatology (Zotev et al., 2018), further highlighting the role of top-down regulatory control over limbic hyperactivity. In contrast, the decreased CEN connectivity observed in this study may reflect the distinct functional demands of PCC regulation, which appear to prioritize recalibration of posterior DMN connectivity over engagement of prefrontal regions within the CEN. Progressively reduced CEN connectivity over the course of neurofeedback training in the PTSD group may signify a gradual shift away from reliance on top-down cortical control from the CEN as participants become more proficient in PCC downregulation over the course of training.

Notably, decreases in CEN connectivity among healthy controls were more modest than in the PTSD group and involved the supramarginal gyrus and inferior frontal gyrus, rather than lateral prefrontal cortex regions typically implicated in emotion regulation, as observed in the PTSD group. Interestingly, a previous analysis using the same dataset revealed unique increased activation in the right dlPFC during regulate compared to view conditions for the control group (Nicholson et al., 2021). While overall CEN connectivity decreased during PCC downregulation, this finding suggests that dlPFC activation may still contribute to regulatory processes in healthy controls, indicating potentially divergent emotion regulation mechanisms in comparison to PTSD participants. This aligns with literature demonstrating significant differences in ICN patterns between individuals with PTSD and healthy controls, particularly during emotion regulation. Indeed, posterior DMN connectivity is often preserved or heightened in PTSD, in contrast to reduced connectivity in prefrontal regions of the DMN and CEN (Akiki et al., 2017, Akiki et al., 2018, Lanius et al., 2015, Nicholson et al., 2018, Rabellino et al., 2015, Shang et al., 2014, Tursich et al., 2015). To further investigate the differential contributions of inter-network (CEN) versus intra-network (DMN) regulation during PCC-targeted fMRI-NFB, we directly compared DMN and CEN connectivity.

### 4.3 DMN versus CEN connectivity

A direct comparison of network connectivity revealed that DMN connectivity exceeded CEN connectivity during regulate compared to view conditions, averaged across all three neurofeedback training runs. In the PTSD group, the DMN demonstrated significantly greater connectivity than the CEN with several regions, including the left occipital pole, left postcentral gyrus, left supramarginal gyrus, right superior frontal gyrus, bilateral middle frontal gyrus, right angular gyrus, and left frontal pole. For the control group, the DMN exhibited significantly greater connectivity than the CEN with the left supramarginal gyrus. Notably, the CEN did not show significantly greater connectivity than the DMN with any regions during regulate compared to view for either group. These findings further support the notion that PCC regulation relies primarily on intra-network dynamics within the DMN rather than inter-network contributions from the CEN, as previously described. Additionally, the broader range of regions showing greater DMN connectivity for the PTSD group in comparison to controls may be attributed to the role of compensatory mechanisms in facilitating emotion regulation. Specifically, it may be that among individuals with PTSD, DMN connectivity becomes more prominent during neurofeedback in response to a reduced capacity to engage CEN-mediated top-down processes, a phenomenon that is well-documented in the literature (Akiki et al., 2017, Lanius et al., 2015, Nicholson et al., 2018, Rabellino et al., 2015, Shang et al., 2014, Tursich et al., 2015).

Taken together, the findings from this study align with our hypothesis that PCC downregulation is primarily associated with intra-network recalibration within the DMN, rather than inter-network contributions from the CEN, particularly in the PTSD group. This conclusion is supported by observations of increased DMN connectivity with regions implicated in PTSD psychopathology, such as the precentral gyrus and anterior insula, among individuals with PTSD and the positive correlations of these connectivity patterns with symptom severity and emotion regulation difficulties. In contrast, the CEN exhibited decreased connectivity across both PTSD and control groups during PCC regulation, with progressively reduced connectivity observed in the PTSD group over the course of neurofeedback training. Furthermore, a direct comparison revealed that DMN connectivity with several brain regions exceeded CEN connectivity during PCC regulation, with no regions showing the opposite pattern; this finding was most pronounced in the PTSD group. Overall, these results support the proposed role of DMN-mediated mechanisms in facilitating PCC downregulation and align with emerging neurobiological evidence highlighting the critical role of the PCC and DMN in emotion regulation processes, particularly within the context of PTSD.

### 4.4 Limitations and future directions

While the present analysis provides valuable insight into the differential contributions of the DMN and CEN to PCC downregulation during fMRI-NFB, several limitations warrant consideration and should be addressed in future research. First, although ICA is effective for identifying patterns of large-scale network connectivity, it provides non-directional measures and cannot determine whether the observed changes reflect excitatory or inhibitory influences. As such, the current findings cannot directly confirm the presence of a recalibration mechanism, even if this remains a plausible interpretation. Future studies should employ effective connectivity methods, such as dynamic causal modelling (DCM). Second, the task-based experimental protocol limited our ability to evaluate whether neurofeedback-mediated changes in connectivity extend beyond the task conditions. Incorporating pre- and post-neurofeedback resting-state scans in future studies would provide a more comprehensive understanding of the broader effects of fMRI-NFB on ICNs, including whether observed changes persist at rest and contribute to long-term symptom improvements. Third, potentially confounding factors, such as socio-economic status (e.g., income level, educational attainment), prior psychotropic medication use, or prior psychotherapy exposure, were not accounted for in the present study but may influence neurofeedback success and ICN connectivity patterns. Future research should collect and control for these variables to enhance the interpretability and generalizability of findings. Fourth, the relatively small sample size limited the ability to examine heterogeneity within PTSD, such as the dissociative subtype, which may exhibit distinct patterns of ICN dysregulation and neurofeedback response. Appropriately powered studies are needed to investigate these subgroups, which could help refine personalized neurofeedback approaches tailored to specific PTSD symptom profiles. Fifth, this study used a single session of fMRI-NFB training, providing important mechanistic insights but not capturing the potential cumulative effects of neurofeedback on ICN connectivity or symptom reduction. Future research should explore the effects of multiple neurofeedback sessions to determine the optimal “dose” required for sustained therapeutic outcomes and robust ICN connectivity changes. Additionally, longitudinal studies examining the durability of connectivity changes and their relationship to clinical improvements over time are critical to understanding the long-term efficacy of neurofeedback in PTSD treatment. This study was not preregistered; preregistration should be prioritized in future work to enhance reproducibility. Additionally, although rigorous artifact correction procedures were applied, we did not quantify or report artifact statistics at the group or condition level, which may be informative for interpreting signal quality and potential confounds.

## 5 Conclusion

This study provides novel insight into the differential contributions of the DMN and CEN in facilitating emotion regulation during PCC-targeted fMRI neurofeedback in individuals with PTSD and healthy controls. Consistent with our hypothesis, PCC downregulation was primarily associated with intra-network recalibration within the DMN, particularly in the PTSD group, as evidenced by increased DMN connectivity with regions implicated in PTSD psychopathology—namely, the precentral gyrus and anterior insula. In contrast, CEN connectivity decreased across both groups during PCC downregulation, with progressively reduced connectivity observed in the PTSD group over the course of neurofeedback training. A direct comparison revealed that DMN connectivity exceeded CEN connectivity with several regions, further emphasizing the unique contributions of DMN-centred processes during neurofeedback. Overall, these findings enhance our understanding of the neural mechanisms underlying PCC-targeted fMRI-NFB in PTSD and highlight the importance of tailoring neurofeedback protocols to leverage network-specific dynamics.

## Funding Information

This research was funded by the Canadian Institute for Veteran Health Research (CIMVHR). Andrew A. Nicholson has received funding support from the European Union's Horizon 2020 research and innovation program under the Marie Sklodowska-Curie Individual Fellowship (grant agreement No. 897709), the Banting Research Foundation (award number 2021–1424) and the Canadian Institutes of Health Research (CIHR) (project grant No. 483268). Jonathan M. Lieberman has received funding support from the Canadian Institutes of Health Research (CIHR) (funding reference No. 187470).

## CRediT authorship contribution statement

**Jonathan M. Lieberman:** Writing – review & editing, Writing – original draft, Visualization, Methodology, Investigation, Formal analysis, Conceptualization. **Ruth A. Lanius:** . **Maria Densmore:** Writing – review & editing, Methodology, Investigation, Data curation. **Jean Théberge:** Writing – review & editing, Software, Methodology. **Daniela Rabellino:** Writing – review & editing, Investigation, Data curation. **Paul A. Frewen:** . **Frank Scharnowski:** Writing – review & editing. **Rakesh Jetly:** Writing – review & editing. **Benicio N. Frey:** Writing – review & editing. **Fardous Hosseiny:** Writing – review & editing. **Tomas Ros:** Writing – review & editing. **Andrew A. Nicholson:** Writing – review & editing, Supervision, Resources, Project administration, Methodology, Investigation, Funding acquisition, Conceptualization.

## Declaration of competing interest

The authors declare that they have no known competing financial interests or personal relationships that could have appeared to influence the work reported in this paper.

## Data Availability

Data will be made available on request.
