# Supplementary figures and images for: Contributions of the default mode and central executive networks during posterior cingulate cortex-targeted fMRI neurofeedback in PTSD
Source: Neuroimage Clin. 2025 Oct 22;48:103891. doi: 10.1016/j.nicl.2025.103891 (PMC12661201; doi:10.1016/j.nicl.2025.103891)

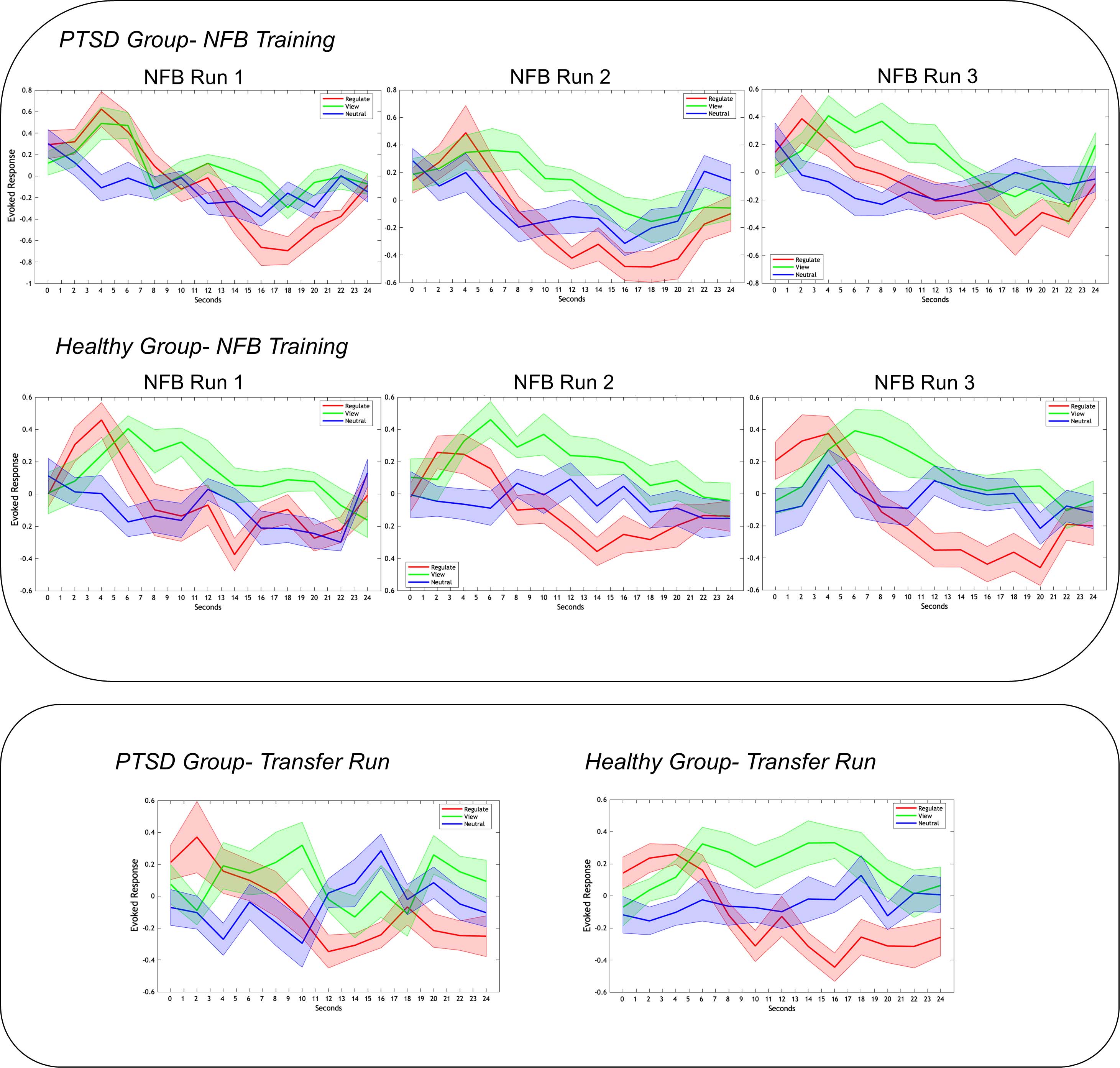

Supplement: Supplementary Fig. 1 [file mmc2.jpg]

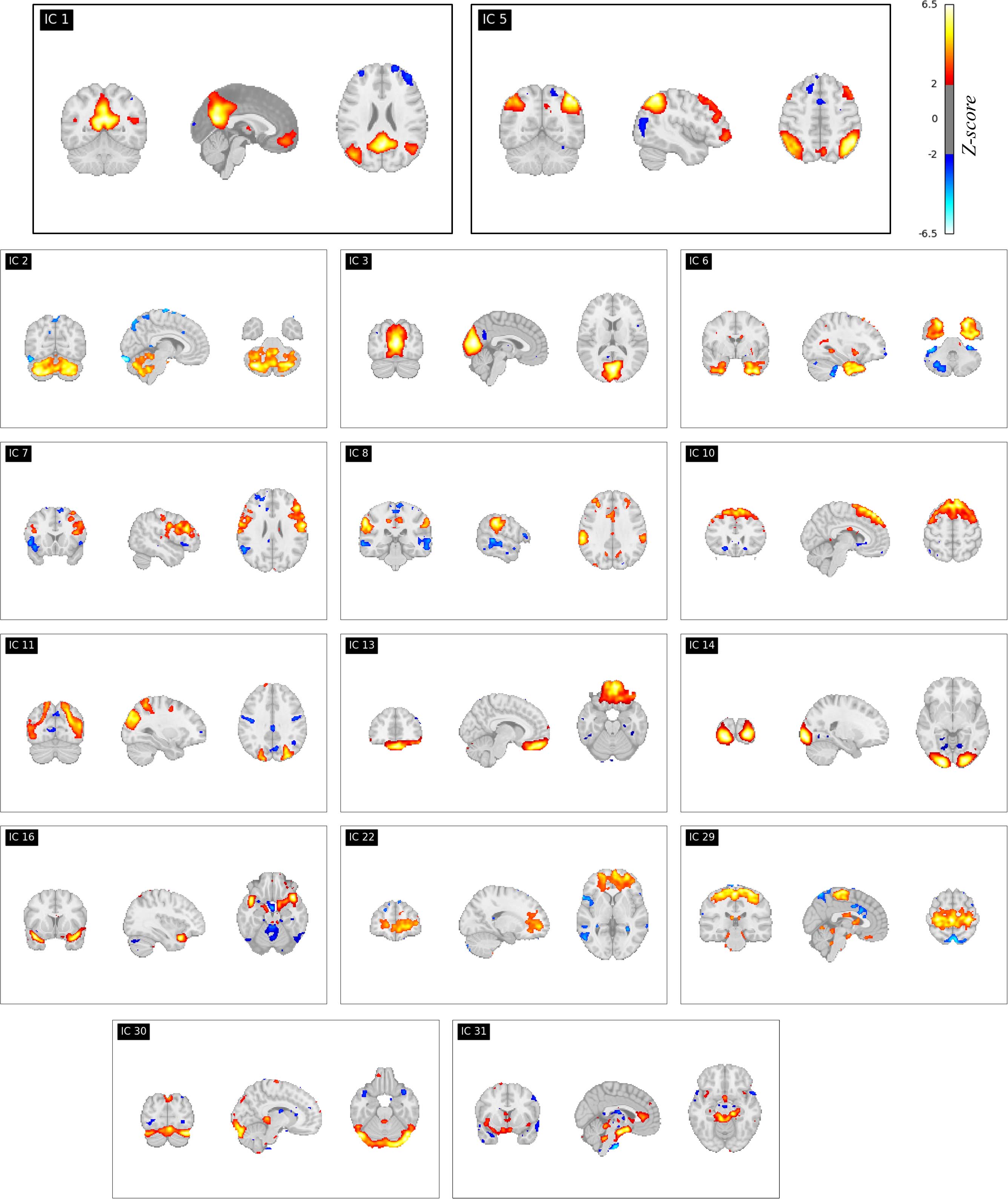

Supplement: Supplementary Fig. 2 [file mmc3.jpg]
