# Supplementary material for: Contributions of the default mode and central executive networks during posterior cingulate cortex-targeted fMRI neurofeedback in PTSD
Source: Neuroimage Clin. 2025 Oct 22;48:103891. doi: 10.1016/j.nicl.2025.103891 (PMC12661201; doi:10.1016/j.nicl.2025.103891)
Supplement: Supplementary Data 1 [file mmc1.pdf]

CRED-nf checklist summary

15 September, 2025

Manuscript title: Contributions of the Default Mode and Central Executive Networks during Posterior Cingulate Cortex-Targeted fMRI Neurofeedback in PTSD
Corresponding Author: Jonathan M. Lieberman
Corresponding author email: liebermj@mcmaster.ca

Item No.	Checklist item	Manuscript Details
**Pre-experiment**		
1a	Pre-register experimental protocol and planned analyses	*This experiment was not preregistered*
1b	Justify sample size	The sample size of this pilot investigation was based on study feasibility during the recruitment period.
**Control groups**		
2a	Employ control group(s) or control condition(s)	Our neurofeedback protocol consisted of three conditions: regulate, view, and neutral. In the regulate condition, participants were instructed to decrease the neurofeedback signal while viewing a personalized trauma-related word (PTSD group) or a matched distressing word (control group). In the view condition, participants viewed the chosen trauma/distressing words but were instructed to respond naturally and not attempt to exert regulatory control over the neurofeedback signal. In the neutral condition, participants viewed a personalized neutral word and were instructed to respond naturally and not attempt to exert regulatory control over the neurofeedback signal.
2b	When leveraging experimental designs where a double-blind is possible, use a double-blind	*NA: A double-blind was not appropriate for this experiment*
2c	Blind those who rate the outcomes	*NA: There was only one participant group*
	Blind those who analyse the data	*NA: There was only one participant group*
2d	Examine to what extent participants and experimenters remain blinded	*NA: There was only one participant group*
2e	In clinical efficacy studies, employ a standard-of-care intervention group as a benchmark for improvement	*NA: This is not a clinical efficacy study*

Control measures		
3a	Collect data on psychosocial factors	Prior to scanning, all participants completed several clinical assessments, including the Beck's Depression Inventory (BDI) (Beck et al., 1997), the Childhood Trauma Questionnaire (CTQ) (Bernstein et al., 2003), Difficulties in Emotion Regulation Scale (DERS) (Gratz & Roemer, 2003), and the Multiscale Dissociation Inventory (MDI) (Briere et al., 2005). After each of the fMRI neurofeedback runs, participants completed the Response to Script Driven Imagery Scale (RSDI) (Hopper et al., 2007), which included the following symptom subscales: reliving, distress, physical reactions, dissociation, and emotional numbing.
3b	Report whether participants were provided with a strategy	Participants were not provided with specific regulation instructions but were told that they would be ”regulating an area of the brain related to emotional experience.”
3c	Report the strategies participants used	Reported in previous publications using this dataset (Lieberman et al., 2023a; Lieberman et al., 2023b). Not repeated here to avoid redundancy.
3d	Report methods used for online-data processing and artifact correction	To present real-time PCC neural activation to participants via a thermometer display, we first imported anatomical scans into BrainVoyager (version QX2.4, Brain Innovations), skull-stripped and transformed them into Talairach space. We then added the normalization parameters into TurboBrainVoyager (TBV, version 3.0, Brain Innovations). TBV was the software used for real-time signal processing and analysis of BOLD signals. During real-time processing, TBV detected and corrected for head movements (using a rigid body transformation) and conducted spatial smoothing using a 4-mm full-width-half-maximum (FWHM) Gaussian kernel. The first two functional scan volumes were removed prior to real-time signal processing.
3e	Report condition and group effects for artifacts	*Condition and group effects for artifacts were not measured, or not reported in the manuscript*
**Feedback specifications**		

4a	Report how the online-feature extraction was defined	The neurofeedback target, the PCC, was defined using a 6 mm sphere at the coordinate (MNI: 0 -50 20) (Bluhm et al., 2009). We used the "best voxel selection" tool in TBV to calculate the BOLD signal amplitude in this target area. This method identifies the 33% most active voxels (i.e., the voxels with the highest beta-values) for the view > neutral contrast. The first two trials of each neurofeedback run were the view and neutral conditions which allowed us to select voxels based on the view > neutral contrast. As outlined in previous publications (Lieberman et al., 2023a, 2023b; Nicholson et al., 2016a, 2018; Paret et al., 2014, 2016), the voxel selection was dynamically updated throughout the duration of training based on (a) the voxel with the largest beta value, and (b) the magnitude of deviation from the mean of all condition betas (Goebel, 2014). This method eliminates inter-subject differences in the number of voxels used for signal extraction while accounting for slight anatomical shifts across runs and/or movement-related slice shifts. In order to create a smoother neurofeedback signal, the mean of the neurofeedback signal of the current and 3 preceding TRs was shown to participants (via thermometer display) (Lieberman et al., 2023a, 2023b; Nicholson et al., 2016a, 2018; Paret et al., 2014, 2016).
4b	Report and justify the reinforcement schedule	In order to create a smoother neurofeedback signal, the mean of the neurofeedback signal of the current and 3 preceding TRs was shown to participants (via thermometer display) (Lieberman et al., 2023a, 2023b; Nicholson et al., 2016a, 2018; Paret et al., 2014, 2016).
4c	Report the feedback modality and content	During neurofeedback training, participants were shown a signal indicating PCC activation. This signal was presented as two thermometers on either side of a screen visible to participants inside the scanner. The bars on the thermometers increased or decreased based on changes in the PCC's BOLD signal. Each segment in the thermometer represented a 0.2% change in PCC activation, with a maximum range of 2.8% increase and 1.2% decrease from baseline. At the start of each trial, the mean of the preceding four data points was taken as the baseline and displayed to participants as an orange line on the thermometer... Before the first run, participants were informed of the delay (~6-8 seconds) in the neurofeedback signal due to the BOLD signal time lag and real-time processing.

4d	Collect and report all brain activity variable(s) and/or contrasts used for feedback, as displayed to experimental participants	The neurofeedback target, the PCC, was defined using a 6 mm sphere at the coordinate (MNI: 0 -50 20) (Bluhm et al., 2009). We used the "best voxel selection" tool in TBV to calculate the BOLD signal amplitude in this target area. This method identifies the 33% most active voxels (i.e., the voxels with the highest beta-values) for the view > neutral contrast. The first two trials of each neurofeedback run were the view and neutral conditions which allowed us to select voxels based on the view > neutral contrast. As outlined in previous publications (Lieberman et al., 2023a, 2023b; Nicholson et al., 2016a, 2018; Paret et al., 2014, 2016), the voxel selection was dynamically updated throughout the duration of training based on (a) the voxel with the largest beta value, and (b) the magnitude of deviation from the mean of all condition betas (Goebel, 2014). This method eliminates inter-subject differences in the number of voxels used for signal extraction while accounting for slight anatomical shifts across runs and/or movement-related slice shifts. In order to create a smoother neurofeedback signal, the mean of the neurofeedback signal of the current and 3 preceding TRs was shown to participants (via thermometer display) (Lieberman et al., 2023a, 2023b; Nicholson et al., 2016a, 2018; Paret et al., 2014, 2016).
4e	Report the hardware and software used	We used the same 3 Tesla MRI Scanner (Siemens Biograph mMR) with a 32-channel head coil at the Lawson Research Institute for all participants...We acquired functional whole-brain images of BOLD contrasts using a gradient echo T2*-weighted echo-planar-imaging sequence (TE=30ms, TR=2s, FOV=192x192mm, flip angle=80°, in-plane resolution=3x3mm). One volume consisted of 36 ascending interleaved slices tilted -20° from the AC-PC orientation. Volumes had a thickness of 3mm and a slice gap of 1mm. The experimental runs comprised 284 volumes each, and T1-weighted anatomical images were obtained using a Magnetization Prepared Rapid Acquisition Gradient Echo sequence (TE=3.03ms, TR=2.3s, 192 slices, FOV=256x256mm). Stimuli were presented using Presentation software from Neurobehavioral Systems. To present real-time PCC neural activation to participants via a thermometer display, we first imported anatomical scans into BrainVoyager (version QX2.4, Brain Innovations), skull-stripped and transformed them into Talairach space. We then added the normalization parameters into TurboBrainVoyager (TBV, version 3.0, Brain Innovations). TBV was the software used for real-time signal processing and analysis of BOLD signals. We preprocessed functional images using SMP12 within MATLAB R2020a.
**Outcome measures - brain**		

5a	Report neurofeedback regulation success based on the feedback signal	Both the PTSD and healthy control groups successfully downregulated PCC activity during regulate compared with view conditions. Detailed statistical analyses of these effects, including repeated measures condition-by-run ANOVAs and between-group comparisons for each condition are reported in (Nicholson et al., (2021); here, we provide a brief summary for context. For both groups, the average event-related BOLD response within the PCC (NFB target region) was significantly lower during regulate than view for each of the three NFB training runs (Figure 2a) as well as the transfer run (Figure 2b). No significant main effect of run or run-by-condition interactions were observed, indicating that PCC downregulation occurred to a similar extent across runs. Additionally, there were no significant differences in BOLD response between the PTSD and healthy control groups during the regulate and view conditions. This implies that both groups were able to successfully downregulate their PCC to a similar extent. However, as observed in prior analyses (Lieberman et al., 2023; Nicholson et al., 2021) as well as current findings (Section 3.3 Spatial comparison), the neural mechanisms supporting this regulation differed substantially between groups. Event-related BOLD responses for all three conditions (neutral, view, regulate
		) are provided in the Supplementary Materials (Figure S1). Methodological details on the event-related BOLD response extraction and calculation—using rfxplot software to generate peristimulus time histograms from the PCC target sphere via a Finite Impulse Response (FIR) model (Gläscher, 2009)—are provided in Nicholson et al. (2021).
5b	Plot within-session and between-session regulation blocks of feedback variable(s), as well as pre-to-post resting baselines or contrasts	See Figures 2 and S1.

5c	Statistically compare the experimental condition/group to the control condition(s)/group(s) (not only each group to baseline measures)	Specifically, for the PTSD compared to the control group, there was significantly increased connectivity between the DMN and the left precentral gyrus and right anterior insula during regulate compared to view conditions, averaged across all three neurofeedback training runs (Figure 3a). Conversely, for both the PTSD and control groups, there were no significant within-group differences in DMN connectivity during regulate compared to view conditions and vice versa, averaged across the neurofeedback training runs. Similarly, for both participant groups, there were no significant within-group changes in DMN connectivity during regulate compared to view conditions, over the course of the neurofeedback training runs (i.e., run three versus run one, and vice versa). In contrast to the DMN, there were no significant between-group differences in CEN functional connectivity. However, for both the PTSD and healthy control groups, there were multiple significant within-group differences in CEN functional connectivity (Table 4). Specifically, the PTSD group showed significantly decreased functional connectivity between the CEN and the left occipital pole, the bilateral middle frontal gyrus, and the right superior frontal gyrus during regulate compared to view conditions, averaged across all three neurofeedback training
		runs (Figure 3b). The control group exhibited a similar pattern with significantly decreased functional connectivity between the CEN and the bilateral supramarginal gyrus and the left inferior frontal gyrus during regulate compared to view conditions, averaged across the training runs (Figure 3b). For the PTSD group, there was progressively decreased CEN connectivity with several brain regions—the right middle occipital/angular gyrus, the right superior parietal lobule, the precuneus/precentral gyrus, the left inferior occipital gyrus, the bilateral supramarginal gyri, the supplementary motor cortex, and the left middle frontal gyrus—during regulate compared to view conditions, over the course of neurofeedback training (i.e., for run three compared to run one) (Figure 4, Table 5). In contrast, no such progressive changes in CEN connectivity were observed for the control group during neurofeedback training.
**Outcome measures - behaviour**		
6a	Include measures of clinical or behavioural significance, defined a priori, and describe whether they were reached	The linear regression analyses revealed significant correlations between DMN functional connectivity and clinical measures during regulate compared to view conditions (Table 7 and Figure 3a). Specifically, connectivity between the DMN and both the left precentral gyrus and right anterior insula showed positive correlations with participant total CAPS scores, indicating a relationship between DMN connectivity and PTSD symptom severity. Additionally, DMN connectivity with the left precentral gyrus was positively correlated with DERS scores, reflecting an association with difficulties in emotion regulation. No significant correlations were found between DMN connectivity and CTQ, BDI, MDI, or RSDI scores. Furthermore, no significant correlations were observed between CEN connectivity and any clinical measures.

6b	Run correlational analyses between regulation success and behavioural outcomes	Reported in previous publications using this dataset (Lieberman et al., 2023a; Lieberman et al., 2023b; Nicholson et al., 2021). Not repeated here to avoid redundancy.
**Data storage**		
7a	Upload all materials, analysis scripts, code, and raw data used for analyses, as well as final values, to an open access data repository, when feasible	*No additional documents related to the materials, analysis scripts, code, raw data, or final values are available for this manuscript*
